# Evaluation of Antimicrobial Usage in Dogs and Cats at a Veterinary Teaching Hospital in Germany in 2017 and 2018

**DOI:** 10.3389/fvets.2021.689018

**Published:** 2021-06-23

**Authors:** Anne Schnepf, Sabine Kramer, Rolf Wagels, Holger A. Volk, Lothar Kreienbrock

**Affiliations:** ^1^Department of Biometry, Epidemiology and Information Processing, WHO Collaborating Centre for Research and Training for Health in the Human-Animal-Environment Interface, University of Veterinary Medicine Hannover, Hannover, Germany; ^2^Department of Small Animal Medicine and Surgery, University of Veterinary Medicine Hannover, Hannover, Germany; ^3^Information and Data Service (TiHo-IDS), University of Veterinary Medicine Hannover, Hannover, Germany

**Keywords:** antimicrobial consumption, electronic practice management software, companion animal, pets, individual animal

## Abstract

In contrast to food-producing animals, where the documentation of the usage of antimicrobials is regulated by law, antimicrobial usage (AMU) in dogs and cats is only sparsely monitored. We collected data generated by an electronic practice management software (EPMS) between January 1, 2017 and December 31, 2018 to investigate AMU. All information was obtained from clinical routine data from the Department of Small Animal Medicine and Surgery (DSAM), University of Veterinary Medicine Hannover (TiHo). In 2017, 78,076 drug administrations were documented for 5,471 dogs and cats, of which 14,020 (17.96%) were antimicrobial drugs (AMs) specifically documented in 2,910 (51.31%) dogs and cats. In 2018, 104,481 drug administrations were documented for 5,939 dogs and cats. Of these drug administrations, 18,170 (17.39%) AM administrations were documented for 3,176 (53.48%) dogs and cats. Despite the increasing documentation of AM administrations, differences between 2017 and 2018 were not statistically significant [odds ratio (OR), 1.01; 95% confidence interval (CI), 0.98–1.03]. Prescription diversity (PD) in 2017 for dogs was 0.92 and for cats 0.89. In 2018, PD for dogs was 0.93 and for cats 0.88. As well as the documented number of AM administrations, the documented amount of active ingredients administered in 2018 (total: 17.06 kg; dogs: 16.11 kg, cats: 0.96 kg) increased compared with 2017 (total: 15.60 kg; dogs: 14.80 kg, cats: 0.80 kg). In 2017 and 2018, the most commonly administered antimicrobial groups were penicillins, nitroimidazoles, and quinolones for dogs and cats, respectively. While the in-house point-of-care administration accounts for the largest share of the documented amount of AMs administered, the highest number of documented AM administrations was assigned to inpatient care in 2017 and 2018, respectively. However, AM administration in outpatient care remained the lowest in both years. Since no statistically significant difference in AM administrations was observed between 2017 and 2018 and the most commonly used AMs at the DSAM were ranked, data can be used as a baseline to evaluate how changes in in-house guidelines and future legal requirements affect the prescribing culture. Data generated within the DSAM should be evaluated annually.

## Introduction

The prescription of antimicrobial drugs (AMs) for bacterial infections has become a standard treatment in human and veterinary medicine. Nowadays, the use of a widespread diversity of antimicrobial classes and active ingredients is common practice, but at the same time, antimicrobial resistance (AMR) is rapidly increasing (1, 2). AMR is a key reason for treatment failure (3) and is responsible for increasing lethality in individuals with otherwise non-lethal infections (4, 5). Therefore, maintaining a selection advantage through the reduction of certain AMs and at the same time monitoring the dosage necessary to establish complete eradication of the infection is crucial for the reduction of AMR.

While the spread of AMR through food-supplying animals has been described thoroughly, companion animals, especially dogs and cats, have been neglected, despite the large amount of documented evidence of cross-species transfer of resistant bacteria between humans and animals (6–8). The probability of transmission through close contact between owners and pets—as these animals are often treated as part of the family and live in the household—is favored. An estimated 13.7 million cats and 7.9 million dogs reside in Germany and are a potential reservoir and vector for resistant bacteria (9).

Particularly with regard to a One Health concept and preserving treatment possibilities, documentation of AM use should be considered a necessity. However, collecting and analyzing data about the usage of antimicrobial drugs in pets has been voluntary so far. The requirement for establishing a documentation system was addressed by the European Union (EU) in the regulation 2019/6 of the European Parliament and of the council of 11 December 2018, which states that data on the use of AMs not only in food-supplying animals but also in pets must be collected and reported (10). This law intended for companion animals specifically will allegedly be passed in 2029 (11).

In Germany, systematically collected and evaluated data on antimicrobial usage (AMU) are unavailable, and only a limited amount of published research exists worldwide regarding the type and amount of AMs used and differences in prescribing practices in dogs and cats. The only reliable data available are annually published reports about data for AM sales requiring a veterinary license in Germany. However, these reports only publish aggregated data per antimicrobial group and do not announce data per animal species or AM. In addition, AMs, which are licensed for humans but used off label under the cascade principle [Medicinal Products Act (AMG) §56a (2) (12)] for animals, are not taken into account.

The use of data from heterogeneous sources for evaluating AMU has been previously reported for clinical routine data (13–17) in different countries and insurance data (18) with different evaluation methods.

The aim of this study was to provide results for antimicrobial usage in dogs and cats at a veterinary teaching hospital in Germany, including quantitative indices for AMU and information on differences in treating dogs and cats. This study is based on previous research on AMU in horses in Germany and uses the same method for data management and analyses (13).

## Materials and Methods

### Data Source

In Germany, AMs for animals must be prescribed by a veterinarian (AMG §56a). As a result, clinical routine data from clinics or practices can be used to evaluate AMU in dogs and cats.

Data were gathered from the Department of Small Animal Medicine and Surgery (DSAM), University of Veterinary Medicine Hannover, Foundation (TiHo). Drugs used within the study period between January 1, 2017 and December 31, 2018 were evaluated. A total of 13,823 dogs and cats were examined over the 2-year study period. At least one drug administration was documented for 11,410 pets, and at least one AM administration was documented for 6,086 pets. These data were generated using an electronic practice management software (EPMS) called easyVet [Veterinärmedizinisches Dienstleistungszentrum (VetZ) GmbH, Isernhagen, Germany]. Data were obtained *via* export from easyVET. Extracted data were provided in Excel format (Microsoft, 2010).

### Cohort, Data Management, and Statistics

Only dogs and cats that had been prescribed at least one drug within the investigated time period were included in the study. For each dog and cat, a unique animal identification number (ID number), breed, gender, date of birth, and all documented weights were reported. For each drug, the following information was collected: treatment date, medicinal product name, amount and unit of the preparation, and whether the drug was administered during the visit or dispensed to the owner. Additional data collected included a unique case ID number and the corresponding categorization of outpatient care, inpatient care, or in-house point-of-care administration (similar to an in-house pharmacy).

For this study, all billed drugs were assumed to be used to treat pets.

**Figure 1:**
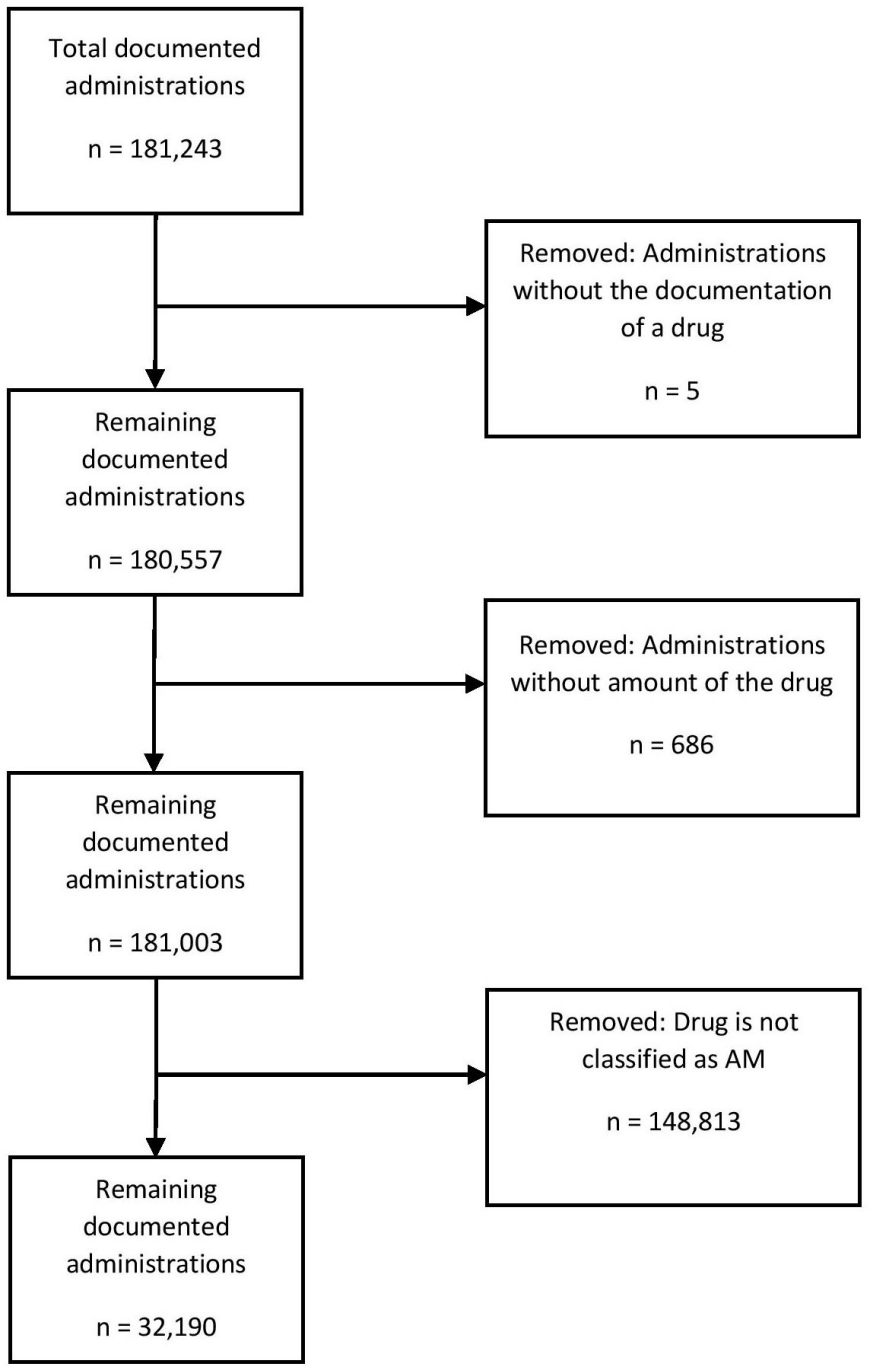
Data cleaning process for drug administrations over the 2-year study period at the Department of Small Animal Medicine and Surgery, University of Veterinary Medicine.

The following prescriptions were excluded (Figure 1): documented administrations without any drug name and documented administrations without any amount of the drug specified.

The master table of drugs used in the study by Schnepf et al. (13) was complemented using the product index of the DSAM. The proportion of active ingredients in each AM and the most recent World Health Organization (WHO) classification for each active ingredient used (*critically important antibiotics* (*CIA)—highest priority, CIA—high priority, highly important*, and *important*; Table 1) were added.

**Table 1:** Active ingredients documented to be used in pets in 2017 and 2018 at the Department of Small Animal Medicine and Surgery, University of Veterinary Medicine Hannover, according to the World Health Organization (WHO) classification, antimicrobial group, and chemical structure.

**WHO classification**	**Antimicrobial group**	**Active ingredient**
CIA^*^–highest priority	Cephalosporins (3rd generation)	Cefixim^1^
		Cefovecin
		Ceftiofur
	Quinolones	Ciprofloxacin^1^
		Enrofloxacin
		Marbofloxacin
		Moxifloxacin^1^
		Ofloxacin^1^
	Macrolides	Spiramycin
		Tylosin
	Polymyxins	Polymyxin B^2^
CIA^*^–high priority	Aminoglycosides	Framycetin^1^
		Gentamicin^2^
		Neomycin^2^
		Spectinomycin
	Penicillins	Amoxicillin^2^
Highly important	Amphenicols	Chloramphenicol^2^
		Florfenicol
	Cephalosporins (1st generation)	Cefalexin
		Cefazolin^1^
	Lincosamides	Clindamycin^2^
		Lincomycin
	Steroid antibacterials	Fusidic acid^2^
	Sulfonamides	Sulfadiazine^2^
		Sulfadoxine
	Tetracyclines	Chlortetracycline
		Doxycycline^2^
	Trimethoprim	Trimethoprim
Important	Nitroimidazoles	Metronidazole^2^
	Polypeptides	Bacitracin

* *CIA, critically important antibiotics*.

1 *drugs exclusively licensed for humans*.

2 *drugs licensed for humans or animals*.

### Calculation of the Administered Daily Amount

The administered daily amount in gram was calculated for each documented drug administration.

$$\begin{array}{l} \text{Administered Daily Amount}\left(ADA\right)=amount\;of\;drug\\ \times proportion\;of\;active\;ingredient\;in\;this\;drug \end{array}$$

For example, if the proportion of amoxicillin in a specific drug in grams per unit was 0.5 (e.g., tablets with 500 mg → 0.5 g/table → proportion of active ingredient in this drug = 0.5) and four tablets were prescribed, then ADA would be 2 g.

### Calculation of the Prescription Diversity

Prescription diversity (PD) was defined by Singleton et al. in 2018 (19) as “the frequency and variety with which a practice prescribes pharmaceutical classes (PC) within a determined pharmaceutical family (PF)” and is calculated as follows:

$$\begin{array}{l} Prescription\;Diversity\;\left(PD\right)=1-\;\frac{\sum np\left(np-1\right)}{NP\left(NP-1\right)}. \end{array}$$

where np = number of prescriptions of a particular PC within a PF and NP the total number of prescriptions within a PF.

For example, amoxicillin is prescribed 300 times, metronidazole 150 times, and cefovecin 200 times.

The equation is as follows

$$\begin{array}{l} PD=1-\;\frac{\left(\left(300\;x\;\left(300-1\right)\right)+\left(150\;x\;\left(150-1\right)\right)+\left(200\;x\;\left(200-1\right)\right)\right)}{650\;x\;\left(650-1\right)}\\ \;=0.64. \end{array}$$

Odds ratios (OR) and 95% confidence interval (CI) were calculated to compare AMU in different groups.

Further methods of data management and statistical calculations are described in the study by Schnepf et al. (13).

All statistical calculations were performed using SAS 9.4M5 (SAS Institute Inc., Cary, NC, USA).

## Results

### Dogs

One hundred thirty-nine thousand nine hundred ninety-four drug administrations were documented for 8,914 dogs with at least one documented drug administration over the 2-year study period. Of these, 24,794 (17.71%) drug administrations were AMs, which were documented for 4,677 (52.47%) dogs. By comparing documented AM administration between 2017 and 2018, the results showed an OR of 1.01 (95% CI, 0.98–1.03).

#### 2017

In 2017, 60,956 drug administrations were documented for 4,383 dogs. The number of documented AM administrations was 10,857 (17.81%) for 2,253 (51.40%) dogs.

Drug administrations were documented for 11,438 unique case ID numbers. AM administrations were documented for 4,232 (37.00%) unique case ID numbers.

The greatest number of AM administrations was documented for inpatient care (*n* = 7,072; 65.14%). Regarding the in-house point-of-care administration, 3,153 (29.04%) AM administrations were documented. The smallest number of AM administrations was documented for outpatient care (*n* = 632; 5.82%).

Most AMs were administered orally (*n* = 6,020; 55.45%), followed by injections (*n* = 4,303; 39.63%) and topical administration (*n* = 534; 4.92%).

The PD for AMs used in 2017 was 0.92.

The three most commonly prescribed AMs were penicillins with amoxicillin (*n* = 6,036; 50.04%) as the only active ingredient used of this AM group, nitroimidazoles with metronidazole (*n* = 2,433; 21.78%), and quinolones (*n* = 709; 6.35%; Table 2).

**Table 2:** Documented number of antimicrobial active ingredients used in dogs and cats in 2017 and 2018 at the Department of Small Animal Medicine and Surgery, University of Veterinary Medicine Hannover.

	**Dogs**			**Cats**		
**Antimicrobial group and active ingredient**	**2017**	**2018**	**Total documented administrations (%)**	**2017**	**2018**	**Total documented administrations (%)**
**Aminoglycoside**	**226**	**189**	**415 (1.63%)**	**22**	**22**	**44 (0.59%)**
Framycetin	1	0	1 (0.00%)	1	0	1 (0.01%)
Gentamicin	133	122	255 (1.00%)	6	11	17 (0.23%)
Neomycin	92	67	159 (0.63%)	14	11	25 (0.34%)
Spectinomycin	–	–	– (–)	1	–	1 (0.01%)
**Penicillins**	**6,036**	**7,562**	**13,598 (53.50%)**	**2,098**	**2,801**	**4,899 (65.77%)**
Amoxicillin	6,036	7,562	13,598 (53.50%)	2,098	2,801	4,899 (65.77%)
**Cephalosporin**	**315**	**388**	**703 (2.77%)**	**51**	**88**	**139 (1.87%)**
Cefalexin	36	16	52 (0.20%)	1	–	1 (0.01%)
Cefazolin	219	247	466 (1.83%)	25	34	59 (0.79%)
Cefixim	23	47	70 (0.28%)	8	13	21 (0.28%)
Cefovecin	5	3	8 (0.03%)	14	9	23 (0.31%)
Ceftiofur	32	75	107 (0.42%)	3	32	35 (0.47%)
**Amphenicol**	**235**	**247**	**482 (1.90%)**	**48**	**46**	**94 (1.26%)**
Chloramphenicol	230	238	468 (1.84%)	46	46	92 (1.24%)
Florfenicol	5	9	14 (0.06%)	2	–	2 (0.03%)
**Quinolones**	**709**	**1,029**	**1,738 (6.84%)**	**200**	**262**	**462 (6.20%)**
Ciprofloxacin	1	8	9 (0.04%)	13	6	19 (0.26%)
Enrofloxacin	113	125	238 (0.94%)	6	5	11 (0.15%)
Marbofloxacin	503	805	1,308 (5.15%)	167	226	393 (5.28%)
Moxifloxacin	5	–	5 (0.02%)	1	0	1 (0.01%)
Ofloxacin	87	91	178 (0.70%)	13	25	38 (0.51%)
**Fusidic acid**	**4**	**4**	**8 (0.03%)**	**1**	**–**	**1 (0.01%)**
Fusidic acid	4	4	8 (0.03%)	1	–	1 (0.01%)
**Lincosamide**	**162**	**178**	**340 (1.34%)**	**51**	**28**	**79 (1.06%)**
Clindamycin	162	178	340 (1.34%)	43	28	71 (0.95%)
Lincomycin	–	–	– (–)	8	–	8 (0.11%)
**Macrolide**	**9**	**19**	**28 (0.11%)**	**–**	**–**	**– (–)**
Spiramycin	7	10	17 (0.07%)	–	–	– (–)
Tylosin	2	9	11 (0.04%)	–	–	– (–)
**Nitroimidazole**	**2,433**	**3,367**	**5,800 (22.82%)**	**560**	**835**	**1,395 (18.73%)**
Metronidazole	2,433	3,367	5,800 (22.82%)	560	835	1,395 (18.73%)
**Polypeptide**	**169**	**200**	**369 (1.45%)**	**25**	**28**	**53 (0.71%)**
Bacitracin	32	26	58 (0.23%)	3	1	4 (0.05%)
Polymyxin B	137	174	311 (1.22%)	22	27	49 (0.66%)
**Sulfonamide**	**216**	**248**	**464 (1.83%)**	**4**	**23**	**27 (0.36%)**
Sulfadiazine	163	197	360 (1.42%)	3	18	21 (0.28%)
Sulfadoxine	53	51	104 (0.41%)	1	5	6 (0.08%)
**Tetracycline**	**440**	**567**	**1,007 (3.96%)**	**118**	**111**	**229 (3.07%)**
Chlortetracycline	2	–	2 (0.01%)	32	26	58 (0.78%)
Doxycycline	438	567	1,005 (3.95%)	86	85	171 (2.30%)
**Trimethoprim**	**215**	**248**	**463 (1.82%)**	**4**	**23**	**27 (0.36%)**
Trimethoprim	215	248	463 (1.82%)	4	23	27 (0.36%)
**Total**	**11,169**	**14,246**	**25,415 (100.0%)**	**3,182**	**4,267**	**7,449 (100.0%)**

“*–” observed zero; “0” zero obtained by rounding. Bold values are the summary per antimicrobial group, the corresponding active ingredients are underneath*.

**Figure 2:**
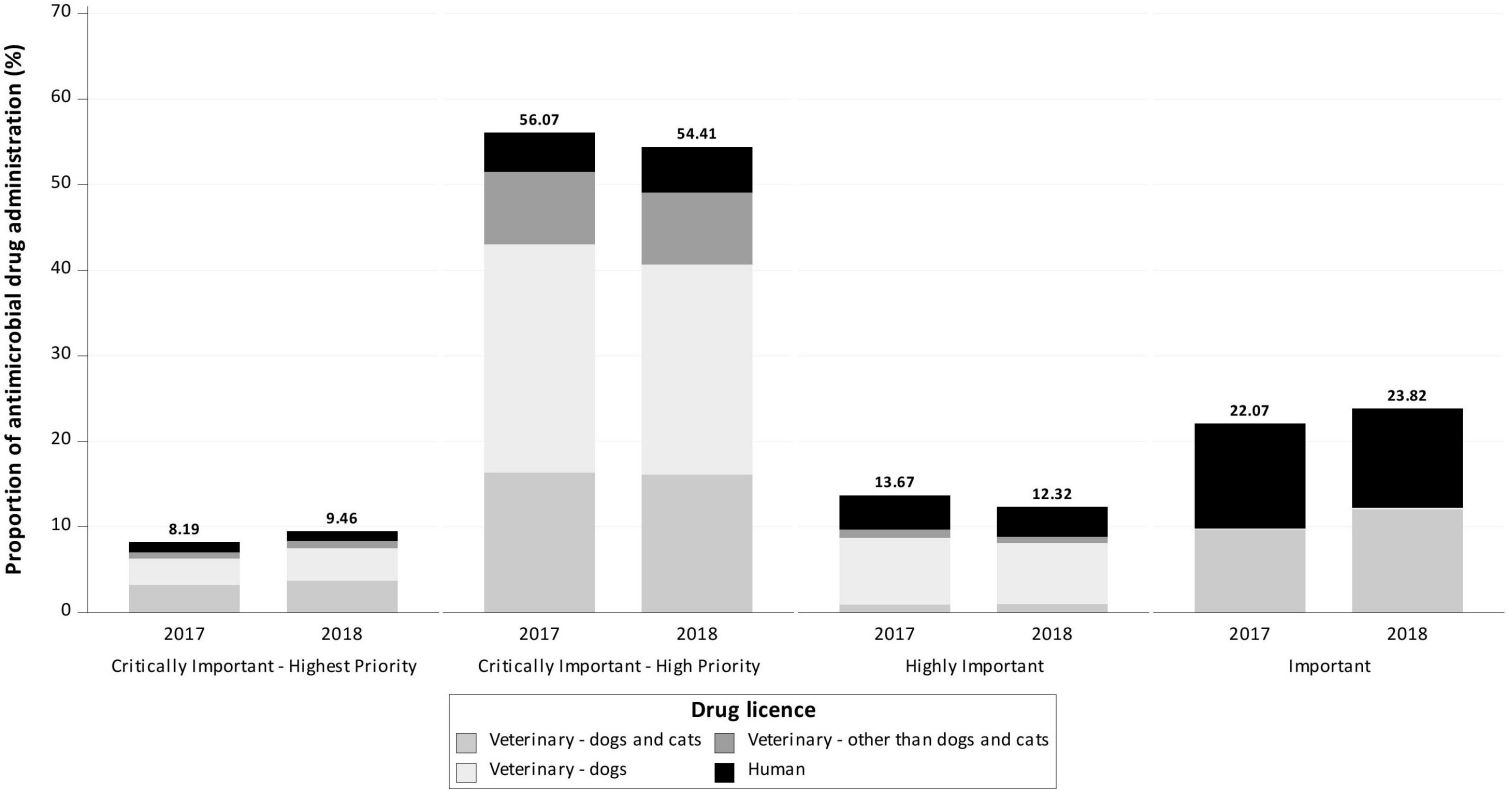
Proportion of documented antimicrobial drug administrations reported to be used in dogs in 2017 and 2018 at the Clinic for Small Animals, University of Veterinary Medicine Hannover, by drug license type and World Health Organization classification.

Notably, 8.19% (*n* = 915) of the active ingredients in the administered AMs were classified by the WHO as CIAs with highest priority, 56.07% (*n* = 6,262) as CIAs with high priority, 13.67% (1.527 kg) as highly important and 22.07% (*n* = 2,465) as important (Figures 2, **4**).

A total of 30.38% (*n* = 3,393) of documented active ingredients were licensed for use in dogs and cats, 37.61% (*n* = 4,201) only for dogs, 10.13% (*n* = 1,131) for animals other than dogs or cats, and 21.88% (*n* = 2,444) for humans.

A total of 490 documented AM administrations were used for preoperative injections in dogs in 2017. Amoxicillin in combination with clavulanic acid (*n* = 228; 46.53%) and cefazolin (*n* = 193; 39.39%) were the most commonly documented AMs (**Table 4**).

Overall, 14.80 kg of active ingredients were documented for dogs in 2017. At 76.76% (11.36 kg), the largest proportion was documented for the in-house point-of-care administration, second for inpatient care at 21.01% (3.11 kg), and last for outpatient care at 2.23% (0.33 kg; Table 3).

**Table 3:** Documented amount of antimicrobial active ingredients used in dogs and cats in 2017 and 2018 at the Department of Small Animal Medicine and Surgery, University of Veterinary Medicine Hannover.

	**Dogs**			**Cats**		
	**2017**	**2018**		**2017**	**2018**	
**Antimicrobial group and active ingredient**	**Amount in kg**	**Amount in kg**	**Total amount in kg (%)**	**Amount in kg**	**Amount in kg**	**Total amount in kg (%)**
**Aminoglycoside**	**0.02**	**0.03**	**0.05 (0.17%)**	**0.01**	**0.00**	**0.01 (0.65%)**
Framycetin	0.00	–	0.00 (0.00%)	0.00	–	0.00 (0.01%)
Gentamicin	0.02	0.03	0.05 (0.16%)	0.00	0.00	0.00 (0.04%)
Neomycin	0.00	0.00	0.00 (0.01%)	0.00	0.00	0.00 (0.03%)
Spectinomycin	–	–	– (–)	0.01	–	0.01 (0.57%)
**Penicillins**	**8.11**	**8.82**	**16.93 (54.79%)**	**0.56**	**0.68**	**1.24 (70.57%)**
Amoxicillin	8.11	8.82	16.93 (54.79%)	0.56	0.68	1.24 (70.57%)
**Cephalosporin**	**0.64**	**0.48**	**1.12 (3.63%)**	**0.02**	**0.01**	**0.03 (1.71%)**
Cefalexin	0.29	0.06	0.36 (1.15%)	0.00	–	0.00 (0.11%)
Cefazolin	0.29	0.26	0.56 (1.80%)	0.00	0.00	0.01 (0.48%)
Cefixim	0.05	0.15	0.20 (0.65%)	0.01	0.01	0.02 (1.04%)
Cefovecin	0.00	0.00	0.00 (0.00%)	0.00	0.00	0.00 (0.05%)
Ceftiofur	0.00	0.01	0.01 (0.03%)	0.00	0.00	0.00 (0.02%)
**Amphenicol**	**0.12**	**0.02**	**0.15 (0.47%)**	**0.01**	**0.00**	**0.01 (0.47%)**
Chloramphenicol	0.12	0.02	0.15 (0.47%)	0.01	0.00	0.01 (0.47%)
Florfenicol	0.00	0.00	0.00 (0.00%)	0.00	–	0.00 (0.00%)
**Quinolones**	**0.17**	**0.16**	**0.33 (1.07%)**	**0.01**	**0.01**	**0.01 (0.78%)**
Ciprofloxacin	0.00	0.00	0.00 (0.00%)	0.00	0.00	0.00 (0.02%)
Enrofloxacin	0.07	0.04	0.11 (0.37%)	0.00	0.00	0.00 (0.05%)
Marbofloxacin	0.10	0.12	0.21 (0.69%)	0.01	0.01	0.01 (0.67%)
Moxifloxacin	0.00	–	0.00 (0.00%)	0.00	–	0.00 (0.00%)
Ofloxacin	0.00	0.00	0.00 (0.01%)	0.00	0.00	0.00 (0.03%)
**Fusidic acid**	**0.00**	**0.00**	**0.00 (0.00%)**	**0.00**	**–**	**0.00 (0.02%)**
Fusidic acid	0.00	0.00	0.00 (0.00%)	0.00	–	0.00 (0.02%)
**Lincosamide**	**0.35**	**0.28**	**0.63 (2.05%)**	**0.03**	**0.01**	**0.04 (2.38%)**
Clindamycin	0.35	0.28	0.63 (2.05%)	0.02	0.01	0.04 (2.00%)
Lincomycin	–	–	– (–)	0.01	–	0.01 (0.38%)
**Macrolide**	**0.15**	**0.04**	**0.19 (0.60%)**	**–**	**–**	**– (–)**
Spiramycin	0.01	0.03	0.04 (0.13%)	–	–	– (–)
Tylosin	0.14	0.01	0.15 (0.47%)	–	–	– (–)
**Nitroimidazole**	**4.22**	**4.99**	**9.21 (29.80%)**	**0.14**	**0.20**	**0.34 (19.24%)**
Metronidazole	4.22	4.99	9.21 (29.80%)	0.14	0.20	0.34 (19.24%)
**Polypeptide**	**0.01**	**0.01**	**0.02 (0.08%)**	**0.00**	**0.00**	**0.00 (0.15%)**
Bacitracin	0.00	0.00	0.01 (0.02%)	0.00	0.00	0.00 (0.02%)
Polymyxin B	0.01	0.01	0.02 (0.06%)	0.00	0.00	0.00 (0.13%)
**Sulfonamide**	**0.43**	**0.64**	**1.07 (3.48%)**	**0.01**	**0.02**	**0.02 (1.36%)**
Sulfadiazine	0.41	0.61	1.02 (3.30%)	0.01	0.02	0.02 (1.33%)
Sulfadoxine	0.02	0.03	0.06 (0.18%)	0.00	0.00	0.00 (0.03%)
**Tetracycline**	**0.48**	**0.50**	**0.98 (3.16%)**	**0.02**	**0.02**	**0.04 (2.41%)**
Chlortetracycline	0.00	–	0.00 (0.00%)	0.00	0.00	0.00 (0.03%)
Doxycycline	0.48	0.50	0.98 (3.16%)	0.02	0.02	0.04 (2.38%)
**Trimethoprim**	**0.09**	**0.13**	**0.21 (0.69%)**	**0.00**	**0.00**	**0.00 (0.27%)**
Trimethoprim	0.09	0.13	0.21 (0.69%)	0.00	0.00	0.00 (0.27%)
**Total**	**14.80**	**16.11**	**30.90 (100.0%)**	**0.80**	**0.96**	**1.75 (100.0%)**

“*–” observed zero; “0” zero obtained by rounding. Bold values are the summary per antimicrobial group, the corresponding active ingredients are underneath*.

#### 2018

A total of 79,038 drug administrations were documented for 4,531 dogs in 2018. The number of documented AM administrations was 13,937 (17.63%) for 2,424 (53.50%) dogs.

Drug administrations were documented for 12,576 unique case ID numbers. AM administrations were documented for 4,822 (38.34%) unique case ID numbers.

The greatest number of AM administrations was documented for inpatient care (*n* = 9,468; 67.93%). Regarding the in-house point-of-care administration, 3,535 (25.36%) AM administrations were documented. The smallest number of AM administrations was documented for outpatient care (*n* = 934; 6.70%).

Oral AM administration was the most common route (*n* = 8,008; 57.46%), followed by injection (*n* = 5,335; 38.28%) and topical AM administration (*n* = 594; 4.26%).

PD for AMs in 2018 (PD = 0.93), and the general treatment pattern were similar to the results from 2017 (Tables 2–4; Figure 2), with some remarkable changes in the AMs used containing quinolones, amoxicillin, or metronidazole as active ingredients (Tables 2, 3; **Figure 4**). Similar numbers and treatment patterns of preoperative injections were observed (Table 4).

**Table 4:** Documented number of antimicrobial active ingredients used as single preoperative injections in dogs and cats in 2017 and 2018 at the Department of Small Animal Medicine and Surgery, University of Veterinary Medicine Hannover.

	**Dogs**			**Cats**		
**Antimicrobial group and active ingredient**	**2017**	**2018**	**Total documented administrations (%)**	**2017**	**2018**	**Total documented administrations (%)**
**Aminoglycoside**	**2**	**1**	**3 (0.28%)**	.	**1**	**1 (0.53%)**
Gentamicin	2	1	3 (0.28%)	.	1	1 (0.53%)
**Penicillins**	**244**	**300**	**544 (50.98%)**	**55**	**53**	**108 (57.14%)**
Amoxicillin	16	17	33 (3.09%)	6	11	17 (8.99%)
Amoxicillin + clavulanic acid	228	283	511 (47.89%)	49	42	91 (48.15%)
**Cephalosporin**	**195**	**228**	**423 (39.64%)**	**22**	**27**	**49 (25.93%)**
Cefazolin	193	227	420 (39.36%)	22	26	48 (25.40%)
Ceftiofur	2	1	3 (0.28%)	.	1	1 (0.53%)
**Lincosamide**	**4**	**1**	**5 (0.47%)**	**3**	.	**3 (1.59%)**
Clindamycin	4	1	5 (0.47%)	3	.	3 (1.59%)
**Nitroimidazole**	**25**	**30**	**55 (5.15%)**	**8**	**12**	**20 (10.58%)**
Metronidazole	25	30	55 (5.15%)	8	12	20 (10.58%)
**Quinolones**	**18**	**15**	**33 (3.09%)**	**2**	**5**	**7 (3.70%)**
Enrofloxacin	3	3	6 (0.56%)	.	.	. (.)
Marbofloxacin	15	12	27 (2.53%)	2	5	7 (3.70%)
**Sulfonamide** **+** **trimethoprim**	**2**	**2**	**4 (0.37%)**	**1**	.	**1 (0.53%)**
Sulfadoxine + trimethoprim	2	2	4 (0.37%)	1	.	1 (0.53%)
**Total**	**490**	**577**	**1,067 (100.0%)**	**91**	**98**	**189 (100.0%)**

*Bold values are the summary per antimicrobial group, the corresponding active ingredients are underneath*.

### Cats

Over the 2-year study period, 40,563 drug administrations were documented for 2,636 cats. Of these, 7,396 (18.23%) drug administrations were AMs, which were documented for 1,409 (53.45%) cats. By comparing documented AM administration between 2017 and 2018, the results showed an OR of 1.01 (KI 0.96–1.06), which is not considered a statistically significant result.

#### 2017

In 2017, 17,120 drug administrations were documented for 1,228 cats. The number of documented AM administrations was 3,163 (18.48%) for 657 (51.01%) cats.

Drug administrations were documented for 2,741 unique case ID numbers. AM administrations were documented for 1,190 (43.41%) unique case ID numbers.

The greatest number of AM administrations was documented for inpatient care (*n* = 2,273; 71.86%). Regarding the in-house point-of-care administration, 752 (23.77%) AM administrations were documented. The smallest number of AM administrations was documented for outpatient care (*n* = 138; 4.36%).

Oral AMU was the most common route of administration (*n* = 1,607; 50.81%), followed by injections (*n* = 1,428; 45.15%) and topical AM administration (*n* = 128; 4.05%).

In 2017, PD for AMs used in cats was 0.89.

The three most commonly prescribed AMs were penicillins with amoxicillin (*n* = 2,098; 65.93%) as the active ingredients of this AM group, nitroimidazoles with metronidazole (*n* = 560; 17.60%) and quinolones (*n* = 200; 6.29%; Table 2).

**Figure 3:**
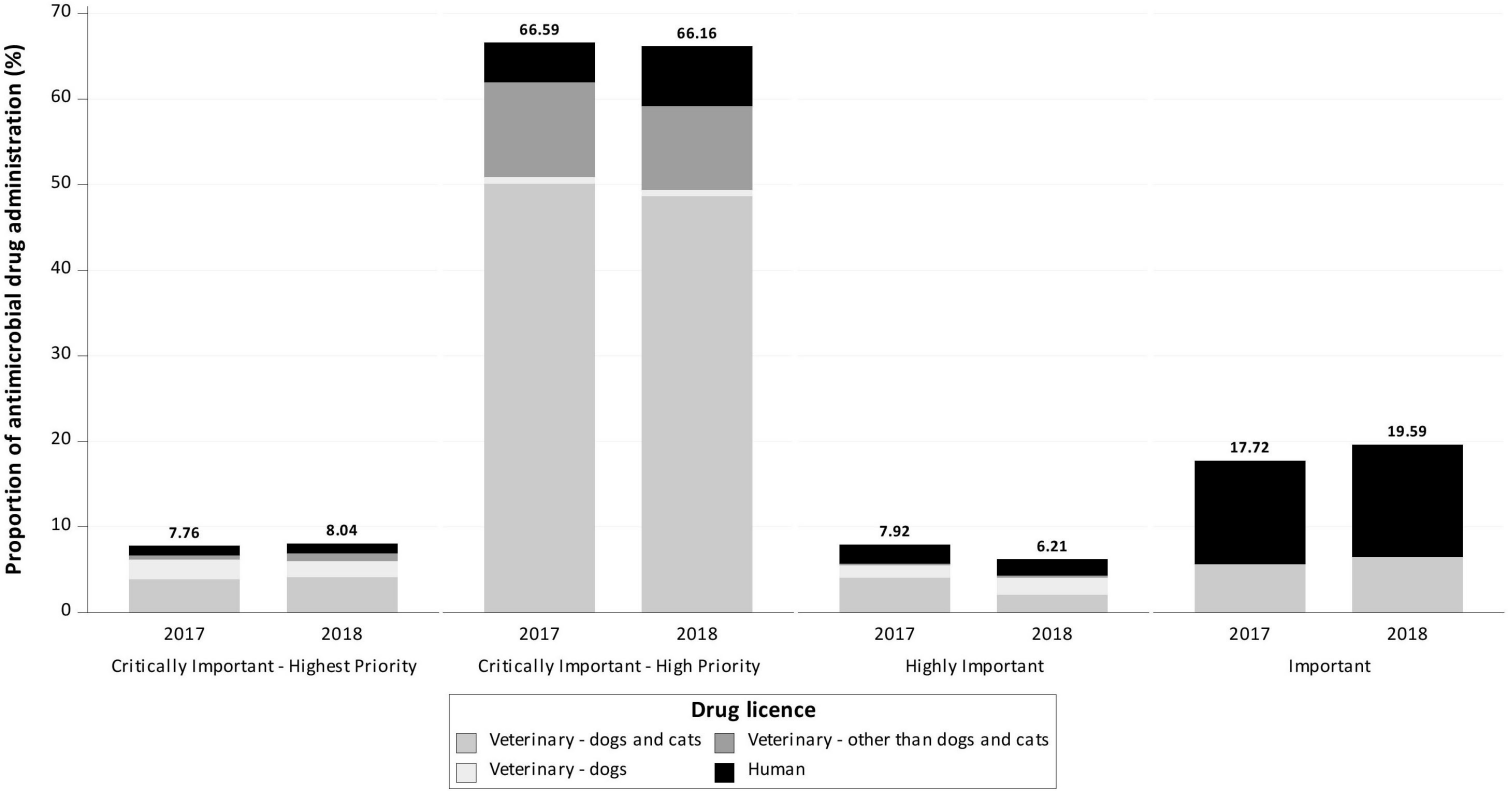
Proportion of documented antimicrobial drug administrations reported to be used in cats in 2017 and 2018 at the Department of Small Animal Medicine and Surgery, University of Veterinary Medicine Hannover, by drug license type and World Health Organization classification.

Notably, 7.76% (*n* = 247) of the active ingredients in AMs were classified by the WHO as CIAs with highest priority, 66.59% (*n* = 2,119) as CIAs with high priority, 7.92% (*n* = 252) as highly important, and 17.72% (*n* = 564) as important (Figures 2, 3).

A total of 63.76% (*n* = 2,029) of documented active ingredients were licensed for use in dogs and cats, 4.68% (*n* = 149) were licensed only for dogs, 11.63% (*n* = 370) were licensed for animals other than dogs or cats and 19.92% (*n* = 634) were licensed for humans.

Ninety-one documented AM administrations were used for preoperative injections in cats in 2017. Amoxicillin in combination with clavulanic acid (*n* = 49; 53.85%) and cefazolin (*n* = 22; 24.18%) were the most commonly documented AMs (Table 4).

Overall, 0.80 kg of active ingredients were documented for cats in 2017. At 73.75% (0.59 kg), the largest proportion was documented for the in-house point-of-care administration, second for inpatient care at 25.00% (0.20 kg), and last for outpatient care at 1.25% (0.01 kg; Table 3).

#### 2018

A total of 23,443 drug administrations were documented for 1,408 cats in 2018. The number of documented AM administrations was 4,233 (16.64%) for 752 (53.41%) cats.

At least one drug administration was documented for 2,994 unique case ID numbers. AM administrations were documented for 1,370 (45.75%) unique case ID numbers.

The greatest number of antimicrobial drug administrations was documented for inpatient care (*n* = 3,190; 75.36%). Regarding the in-house point-of-care administration, 859 (20.29%) drug administrations were documented. The smallest number of antimicrobial drug administrations was documented for outpatient care (*n* = 184; 4.35%).

Oral AMU was the most common administration route (*n* = 2,212; 52.26%), followed by injections (*n* = 1,890; 44.65%) and topical AM administrations (*n* = 131; 3.09%).

**Figure 4:**
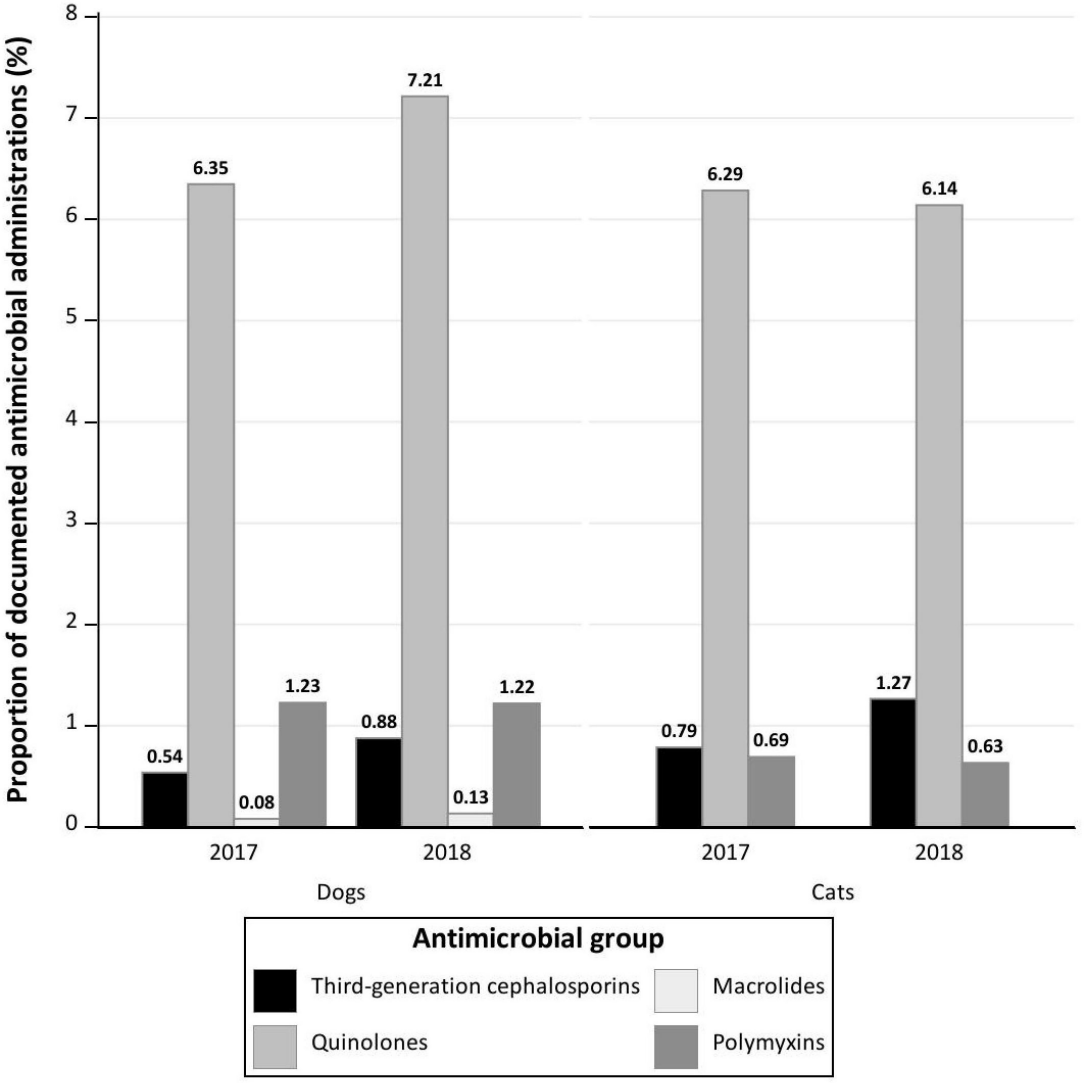
Proportion of documented antimicrobial applications of Critically Important Antibiotics (CIA)—Highest Priority for dogs and cats over the 2 year study period at the Department of Small Animal Medicine and Surgery, University of Veterinary Medicine Hannover.

Equivalent to dogs, the general treatment pattern in cats in 2018 was similar to that in 2017 (Tables 2–4; Figures 3, 4) and had a PD of 0.88. Changes in AMs used were equivalent to findings in dogs, with an increased number of documented AM administrations of quinolones, metronidazole, and amoxicillin. Similar results were obtained for the number and treatment pattern of preoperative injections (Table 4).

## Discussion

With the increasing threat of antimicrobial resistance and with the aim of preserving treatment possibilities, longitudinal data on AMU must be collected, which has become accessible by the increasing percentages of EPMSs used as documentation tools. In particular, due to the updated legal requirements in the EU since 2019, changes in documenting AMU, including assessments of prescription quantities and data on the AMs used, have become a focus. Reporting AMU in dogs and cats will become mandatory at the beginning of January 2029 (10, 11). In addition, the European Medicine Agency (EMA) (11) advises the EU to include AMs licensed for humans in documentation to address the increasing threat of AMR.

To date, a system for the collection and evaluation of data on the use of antimicrobials and qualitative and quantitative indices of AMU in pets in Germany has not been developed. This study aims to elucidate the quantity and antimicrobial classes used in pets at TiHo in 2017 and 2018 and is based on a system that was established by Schnepf et al. (13) for evaluating data from this particular EPMS regarding AMU. The only reliable information that can be used for the comparative analysis is annually published sales data for AMs, which cannot be traced to a specific animal species due to multiple authorizations (20).

The results of this study are not representative of AMU in all clinics and practices in Germany, as the investigated clinic is a multispecialty teaching hospital in a University setting. These areas of technical and personal expertise may lead to a selection bias with a presentation of more severe cases requiring a higher level of AMU. On the other hand, no scientific data are available from other sources in Germany. Therefore, the prescribed results might serve as a baseline for AMU discussion in Germany and across the EU.

Overall, 180,557 documented drug administrations were investigated over a 2-year period. Of these, 32,190 (17.83%) were documented drug administrations with drugs containing at least one AM. These numbers are slightly higher than findings from Australia (16) but lower than findings from Italy (21) and comparable with findings from the UK (14).

Although this study showed a slight decrease in the proportion of prescribed AMs, the differences in the proportion of prescribed AMs between 2017 and 2018 (OR, 1.01; 95% CI, 0.98–1.03) and between dogs and cats in 2017 (OR, 0.97; 95% CI, 0.92–1.01) and 2018 (OR, 0.97; 95% CI, 0.94–1.01) were not statistically significant, respectively. The increasing number and amount of documented AM administrations are associated with the increasing number of animals treated. In addition, the most commonly prescribed AM groups were the same for dogs and cats in 2017 and 2018, respectively, and remained the same between 2017 and 2018.

Drugs containing amoxicillin as the active ingredient were mainly prescribed, which has been previously described in a survey by De Briyne et al. (22), for dogs and cats in Australia (16, 18), the UK (14, 23), and Italy (21) and dogs in the USA (24). This result was expected, as amoxicillin is used as the first-line AM and has only little adverse effects (25). In the present study, metronidazole was the second most commonly documented AM, with quinolones coming in third, which contradicts other findings, in which cephalosporins are more frequently prescribed and metronidazole is prescribed rather infrequently (14, 16). Quinolones, as topoisomerase inhibitors, show an increasing rate of resistance, while metronidazole may produce severe side effects in cats and dogs (26–29); therefore, prudent use is essential. In this study, we did not link corresponding diagnoses with prescribed AMs; therefore, it is possible that the indication for prescribing metronidazole could be to treat also parasites and not bacterial infections alone. For more detailed evaluations and comprehensive results regarding the use of active ingredients, linkage with corresponding diagnoses would be needed.

The range of active ingredients prescribed overall is also similar to the range of active ingredients used for single preoperative injections in dogs and cats in 2017 and 2018, respectively. The prophylactic use of AMs may exert a positive effect on the frequency of surgical site infections in humans (30–32) and animals (33–35), but the time between the injection and first incision is crucial (35, 36). In general, apart from perioperative AMUs, guidelines have been established to reduce the systemic administration of AMs (37, 38). AMU should be limited and targeted to treat an active infection rather than trying to prevent a potential future infection.

While other publications, such as the studies by Singleton et al. (14, 19), Schmitt et al. (39), Escher et al. (21), Wayne et al. (24), and Hur et al. (16), identify cephalosporins as the most widely used AMs, cephalosporins were rarely prescribed in our study, accounting for 2.56% of all prescribed AMs in 2017 and 2018. Only 1st- and 3rd-generation cephalosporins were documented, with 1st-generation cephalosporins (68.65%) displaying the most frequent administration. In particular, cefovecin, which is used increasingly in cats in the UK (14, 17), was infrequently administered in this study (*n* = 31) and its administration to cats and dogs even completely ceased in this study period over time.

In Germany, antimicrobial susceptibility testings are required for dogs and cats if the antimicrobial is not licensed for the treated animal species or for the usage of fluoroquinolones or 3rd- and 4th-generation cephalosporins since March 1, 2018 (40). Further research is required to investigate whether legal changes will result in decreasing usage of critically important antibiotics (CIA)—highest priority.

The highest number of documented AM administrations applies to inpatient care and the lowest number applies to outpatient care. These results were expected, as the clinic is specialized and cares for severely sick patients requiring hospitalization. Routine health checks and vaccinations are not standard treatment procedures performed in this clinic.

However, for both animal species treated in our study, the proportion of AMs licensed for humans was ~20% of administered AMs, with cats having a slightly higher proportion of AMs licensed for humans than dogs. This finding differs from the results reported by Singleton et al. (19), where only 11.4% of prescribed AMs in dogs and 5.7% in cats were authorized for humans, while total AM drug administration was approximately the same for dogs and lower for cats. Compared with our study, Singleton et al. investigated a sentinel network of 457 clinics and practices all over the UK. The relatively high proportion of AMs used under the cascade in the study clinic might be due to selection bias. It could be that drugs licensed for humans were used under the cascade if no alternative with the appropriate route of administration was available. Therefore, the active ingredients used in combination with the route of administration and corresponding diagnosis should be investigated further. Overall, the use of sales data for veterinary drugs alone would therefore not provide an accurate picture of AMU in dogs and cats in Germany.

Singleton et al. (19) described a significant increase in PD for AMs in dogs (PD, 0.83) compared with cats (PD 0.75), which was also reflected in our study. In general, PD was higher than PDs reported by Singleton et al., but PD for dogs was higher than PD for cats in 2017 (0.92 vs. 0.89) and 2018 (0.93 vs. 0.88). While a greater number of AMs were used in dogs, this distribution of AMU is more homogeneous. In our study, a greater number of dogs were treated than cats, which may reflect differences in diseases between species. PD must be calculated for each predefined group, including the organ systems affected, etiology, or selected diseases, to obtain a more detailed comparison.

As shown in our study, the method described for horses (13) is transferable to investigations of AMU in other animal species as well. As a statistically significant difference between the proportion of antimicrobial drug administration in 2017 and 2018 was not observed, the results from these years can serve as a baseline for monitoring AMU in this clinic. Further investigations are needed to evaluate the effect of changes in legal requirements and in-house guidelines for AMU. The most recent indices on AMU can be compared with the indices in this study, and changes in prescribing habits, frequency of AMs used, and type of AMs used can be visualized.

Based on the results from our study, we are able to provide a report at the clinic level, but further investigations are necessary to report usage per individual clinician or service to provide more detailed feedback on AMU and guidelines, which may have a positive influence on AMU (41).

## Conclusions

In this study, we showed that an evaluation of longitudinal data is necessary to appraise changes in prescribing practice and the effects of legal regulation.

Evidently, the method used for horses described by Schnepf et al. (13) in 2020 can be used for pets as well with only minimal adjustments. Therefore, data from other animal species generated with this particular EPMS can be assessed with few modifications for the animal species and drugs used.

In general, data generated with EPMS provide a comprehensive picture of AMU, including AMs licensed for humans, a practice promoted by the EU. A representative of the collective data must be established to represent the AMs used in pets in Germany. Basic standards were determined in this study that can be used universally across platforms and species to ensure comparability of data.

The results of this evaluation will be used as a baseline for AMU in the DSAM. Any prospective research will be compared with this baseline and will be used to evaluate changes in AMU and in-house guidelines.

## Data Availability Statement

The datasets presented in this article are not readily available because the data were available through University intern cooperation. Therefore, any data transfer to interested persons is not allowed without an additional formal contract. Data are available to qualified researchers who sign a contract with the University of Veterinary Medicine Hannover. This contract will include guarantees to the obligation to maintain data confidentiality in accordance with the provisions of the German data protection law. Currently, there exists no data access committee or another body who could be contacted for the data. But for this purpose, a committee will be founded. This future committee will consist of the authors as well as members of the University of Veterinary Medicine Hannover. Interested cooperative partners, who are able to sign a contract as described above, may contact: LK, Department of Biometry, Epidemiology and Information Processing University of Veterinary Medicine, Hannover Bünteweg 2, 30559 Hannover Email: lothar.kreienbrock@tiho-hannover.de. Requests to access the datasets should be directed to lothar.kreienbrock@tiho-hannover.de.

## Ethics Statement

Data used within this study are based on data generated for accounting and documentation purposes. Our research does not involve any regulated animals, and no scientific procedures were performed on animals of any kind. For this reason, formal approval by an ethics committee was not necessary under the provisions of the German regulations.

## Author Contributions

AS and LK: conceptualization, formal analysis, investigation, methodology, and writing—original draft. AS and RW: data curation. AS: project administration, software, and validation. LK: supervision. AS, RW, SK, HV, and LK: writing—review and editing. All authors contributed to the article and approved the submitted version.

## Conflict of Interest

The authors declare that the research was conducted in the absence of any commercial or financial relationships that could be construed as a potential conflict of interest.

## Abbreviations

ADA: administered daily amount
AM(s): antimicrobial drug(s)
AMG: Medicinal Products Act (“Arzneimittel-Gesetz”)
AMR: antimicrobial resistance
AMU: antimicrobial usage
CI: confidence interval
DSAM: Department of Small Animal Medicine and Surgery
CIA(s): critically important antibiotic(s)
PC: pharmaceutical class
PD: prescription diversity
PF: prescription family
EMA: European Medicines Agency
EPMS: Electronic Practice Management Software
EU: European Union
ID number: identification number
OR: odds ratio
TiHo, University of Veterinary Medicine Hannover: Foundation (Stiftung Tierärztliche Hochschule Hannover)
WHO: World Health Organization.
